# Disinfection efficacy of Oxivir TB wipe residue on severe acute respiratory coronavirus virus 2 (SARS-CoV-2)

**DOI:** 10.1017/ice.2023.140

**Published:** 2023-11

**Authors:** Amanda M. Graves, Aaron Barrett, Bechtler Addison, Christopher R. Polage, Becky A. Smith, Sarah Lewis, Deverick J. Anderson, Bobby G. Warren, for the CDC Prevention Epicenters Program

**Affiliations:** 1 Duke Center for Antimicrobial Stewardship and Infection Prevention, Durham, North Carolina; 2 Division of Infectious Diseases, Duke University Medical Center, Durham, North Carolina; 3 Disinfection, Resistance, Transmission and Epidemiology Laboratory, Department of Medicine, Duke University Medical Center, Durham, North Carolina

## Abstract

We assessed Oxivir Tb wipe disinfectant residue in a controlled laboratory setting to evaluate low environmental contamination of SARS-CoV-2. Frequency of viral RNA detection was not statistically different between intervention and control arms on day 3 (P=0.14). Environmental contamination viability is low; residual disinfectant did not significantly contribute to low contamination.

Severe acute respiratory syndrome coronavirus 2 (SARS-CoV-2) environmental contamination has been studied and assessed using reverse-transcriptase polymerase chain reaction (RT-PCR) on various surface materials and survival times.^1–5,7–10^ During a previous study, we visually observed persistent residue build-up from Oxivir TB wipes on the surface of high-touch areas in COVID-19 patient rooms. In this study, 5.5% of surfaces were positive by RT-PCR, and only 0.3% were positive by cell culture.^5^ Concurrent with low environmental contamination of SARS-CoV-2, we hypothesized that disinfectant wipe residue may have influenced our low positivity rates of high-touch areas in COVID-19 patient rooms.

## Methods

We performed an experimental analysis in a laboratory setting of disinfection efficacy of residue from a hydrogen-peroxide–based disinfectant wipe listed on US Environmental Protection Agency (EPA) List N for use against emerging viral pathogens and tested against SARS-CoV-2 called Oxivir TB wipes, (Diversey, Fort Mill, SC). Two materials were used to mimic surfaces commonly used in hospital rooms: formica and stainless steel. Surfaces were assigned cycle threshold (Ct) values from COVID-19 patient samples, initially processed for diagnostic purposes, and a study arm: intervention and control.

In the intervention arm, a study member applied 1 disinfectant wipe to each sterile 10-cm ×10-cm surface, thoroughly drenched, and allowed to air dry for ∼1 hour (study day 1). The control arm received no intervention. All samples remained in a biosafety cabinet under ambient white light at 22°C, mimicking patient rooms. On study day 1, after the disinfectant completely dried, surfaces were inoculated with 50 µL of COVID-19 samples with known Ct values in 6.5-cm diameter predrawn circles.

Each sheet was sampled 48 hours after inoculation (study day 3) with a flocked nylon swab premoistened with viral transport media. RNA extractions were then performed using the Qiagen viral RNA extraction kit (Qiagen, Germantown, MD) and were assessed for the presence of the SARS-CoV-2 N1 gene according to the CDC 2019 novel coronavirus real-time RT-PCR diagnostic panel.^6^

The Z-score proportionality test was used to compare Ct values in positive samples, and the Wilcoxon ranked-sum test was used to compare positive samples between different surfaces and study days. All statistical tests were 2-tailed, and a *P* value <.05 was considered statistically significant. The data analysis was generated using SAS software (SAS Institute, Cary, NC).

## Results

From July 2021 to March 2022, 40 clinical samples were used to inoculate the surfaces on study day 1. In total, 261 sample surfaces were inoculated on day 1 and sampled on day 3. All samples were positive on day 1, and 143 (54.8%) were positive on day 3. The overall median Ct values on study days 1 and 3 were 20.4 (IQR, 16.5–29.8) and 27.3 (IQR, 23.7–31.8), respectively. The median Ct values on the intervention and control arms on day 1 were 18.6 (IQR, 14.7–25.7) for formica and 24.5 (IQR, 17.8–32.0) for stainless steel. The median Ct values on day 3 for the study arms increased for both surfaces. We compared median Ct values for all formica areas (*P* = .04) and all stainless-steel areas (*P* < .001) from day 1 to day 3. However, the frequency of viral RNA detection was not statistically different between the intervention and control arms on day 3 (*P* = .14).

Compared with the formica control arm Ct value of 24.1 (IQR, 22.9–31.1) on day 3, the median Ct value for the intervention arm was 25.7 (IQR, 23.6–31.9; *P* = .17). Similarly, compared with the stainless-steel control arm CT value of 27.3 (IQR, 24.8–32.0) on day 3, the median Ct for the intervention arm was 28.3 (IQR, 25.5–32.3; *P* = .52) (Table 1).

**Table 1.** Median Cycle Thresholds and Proportion of Positive Samples by Study Arm, Study Day, and Sample Material

Sample Day and Material	Intervention	Control	*P* Value
Ct values	Median (IQR)	Median (IQR)	Intervention vs Control
**Sample Day 1**	N = 175	N = 86	
Total	20.4(16.5–29.8)	20.4(16.6–29.7)	
Formica	18.6(14.7–25.7)	18.6(14.7–25.7)	
Stainless steel	24.5(17.8–32.0)	24.5(17.8–32.0)	
**Sample Day 3**	N = 175	N = 86	
Total	27.6(24.0–32.2)	25.8(23.4–31.4)	
Formica	25.7(23.6–31.9)	24.1(22.9–31.1)	
Stainless steel	28.3(25.5–32.3)	27.3(24.8–32.0)	
**RT-PCR positive sample**	No. (%)	No. (%)	
	N=350	N=172	
Total	267 (76)	137 (80)	.67
Formica	138 (39)	71 (41)	.75
Stainless steel	129 (37)	66 (38)	.77
**Sample Day 1**	N=175	N=86	
Total	175 (100)	86 (100)	.99
Formica	87 (50)	42 (49)	1.0
Stainless steel	88 (50)	44 (51)	1.0
**Sample Day 3**	N=175	N=86	
Total	91 (52)	52 (60)	.14
Formica	51 (29)	29 (34)	.17
Stainless steel	41 (23)	22 (26)	.52

Note: IQR, interquartile range; Ct, cycle threshold; RT-PCR, reverse-transcriptase polymerase chain reaction.

## Discussion

In our experiment, Ct values increased and samples with detectable levels of SARS-CoV-2 decreased for the study arms; however, these differences were not statistically significant between arms. These findings suggest that our hypothesis was incorrect; disinfectant residue did not significantly contribute to low contamination rates of surfaces.

Our results are novel because few studies have examined the potential contributors other than natural virion degradation to low environmental contamination of SARS-CoV-2. In general, our results are similar to those of previous studies, which reported that most healthcare environments were negative for SARS-CoV-2 contamination.^7,8^ In contrast, several studies reported SARS-CoV-2 persistence on similar nonporous surfaces in experimental settings for up to 3–7 days; however, both experimental studies did not study SARS-CoV-2 after the use of disinfectants.^4,9,10^

Our study had several limitations. First, repeat sampling was performed which may have inflated decreasing values because we removed virions from the surfaces. Second, only 2 types of surfaces were included; thus, our results may not be generalized to other surface types. Third, this was a controlled laboratory experiment, which can yield lower external validity. As a result, generalizability to frequently disturbed surfaces in patient rooms may be limited. Fourth, convenience sampling from patient specimens may not be fully representative of SARS-CoV-2–positive patients but may be more generalizable than strictly experimental conditions with prespecified Ct values. Finally, Oxivir TB wipes are a disinfectant wipe that kills pathogens, including SARS-CoV-2, on contact, but does not claim to have any long-term impact. However, this type of experiment allowed us to observe the effects of residual disinfectant rather than its long-term activity.

In conclusion, our results suggest that Oxivir TB wipes did not have obvious residual anti-viral activity against SARS-CoV-2 on formica or stainless steel between study arms. By contextualizing it, our results provide evidence that SARS-CoV-2 environmental contamination viability is low; thus, addressing the importance of disinfection strategies in healthcare settings, especially when combatting SARS-CoV-2. Future studies are needed to determine the importance of mitigating the risk of surface contamination of SARS-CoV-2.
